# High-resolution soft X-ray beamline ADRESS at the Swiss Light Source for resonant inelastic X-ray scattering and angle-resolved photoelectron spectroscopies

**DOI:** 10.1107/S0909049510019862

**Published:** 2010-07-10

**Authors:** V. N. Strocov, T. Schmitt, U. Flechsig, T. Schmidt, A. Imhof, Q. Chen, J. Raabe, R. Betemps, D. Zimoch, J. Krempasky, X. Wang, M. Grioni, A. Piazzalunga, L. Patthey

**Affiliations:** a Swiss Light Source, Paul Scherrer Institut, CH-5232 Villigen-PSI, Switzerland; b Paul Scherrer Institut, CH-5232 Villigen-PSI, Switzerland; c Institut de Physique de la Matiére Condensé, Ecole Polytechnique Fédéderale de Lausanne, Switzerland; d Dipartimento di Fisica, Politecnico di Milano, Piazza Leonardo da Vinci 32, I-20133 Milano, Italy

**Keywords:** Swiss Light Source, fixed-gap undulator, soft X-ray beamline, X-ray optics, plane-grating monochromator, grating optimization, ellipsoidal refocusing optics, resonant inelastic X-ray scattering, angle-resolved photoelectron spectroscopy

## Abstract

The concepts and technical realisation of the high-resolution soft X-ray beamline ADRESS operating in the energy range from 300 to 1600 eV and intended for resonant inelastic X-ray scattering (RIXS) and angle-resolved photoelectron spectroscopy (ARPES) are described. The photon source is an undulator of novel fixed-gap design where longitudinal movement of permanent magnetic arrays controls not only the light polarization (including circular and 0–180° rotatable linear polarizations) but also the energy without changing the gap. The beamline optics is based on the well established scheme of plane-grating monochromator operating in collimated light. The ultimate resolving power *E*/Δ*E* is above 33000 at 1 keV photon energy. The choice of blazed *versus* lamellar gratings and optimization of their profile parameters is described. Owing to glancing angles on the mirrors as well as optimized groove densities and profiles of the gratings, the beamline is capable of delivering high photon flux up to 1 × 10^13^ photons s^−1^ (0.01% BW)^−1^ at 1 keV. Ellipsoidal refocusing optics used for the RIXS endstation demagnifies the vertical spot size down to 4 µm, which allows slitless operation and thus maximal transmission of the high-resolution RIXS spectrometer delivering *E*/Δ*E* > 11000 at 1 keV photon energy. Apart from the beamline optics, an overview of the control system is given, the diagnostics and software tools are described, and strategies used for the optical alignment are discussed. An introduction to the concepts and instrumental realisation of the ARPES and RIXS endstations is given.

## Abstract 2

Concepts and technical realization of the high-resolution soft X-ray beamline ADRESS at the Swiss Light Source are described. Optimization of the optical scheme for high resolution and photon flux as well as diagnostics tools and alignment strategies are discussed.

## 1. Introduction

Spectroscopic experiments based on the interaction of light with condensed matter belong to the most important tools in contemporary solid state physics. Light in the spectral regions of vacuum ultraviolet and soft X-rays is particularly sensitive to the electronic properties owing to its strong coupling to valence electrons in the solid. The advanced resonant spectroscopy (ADRESS) beamline is a high-resolution undulator beamline constructed for resonant inelastic X-ray scattering (RIXS) and angle-resolved photoelectron spectroscopy (ARPES) experiments in the soft X-ray region. The beamline is installed at the Swiss Light Source (SLS) on the site of Paul Scherrer Institut, Villigen, Switzerland. SLS is a third-generation synchrotron radiation source with an extremely stable storage ring operating in top-up mode with an electron energy of 2.4 GeV and current of 400 mA. The ADRESS beamline occupies one of nine straight sections of SLS available for insertion devices.

The ADRESS beamline delivers soft X-ray radiation with the following parameters: (i) photon energy range 300 to 1600 eV; (ii) circular and linear polarizations, with the linear polarization vector variable within 0–180°; (iii) ultimate resolving power *E*/Δ*E* above 33000 at 1 keV photon energy; (iv) photon flux at the sample (depending on the operational mode) between 3 × 10^11^ and 1 × 10^13^ photons s^−1^ (0.01% BW)^−1^ at 1 keV photon energy; (v) vertical × horizontal FWHM spot size on the sample below 3.9 µm × 52 µm for the RIXS endstation and 10 µm × 73.6 µm for the ARPES endstation.

The scientific profile of the beamline is focused on strongly correlated electron systems such as transition metal and rare earth oxides. These systems are nowadays at the forefront of solid state physics owing to a variety of their fascinating and practically important properties such as high-temperature superconductivity, metal–insulator transitions, colossal magnetoresistance, *etc*. High-resolution RIXS and ARPES available at the ADRESS beamline are among the most powerful experimental techniques for studying strongly correlated systems. ARPES experiments in the soft X-ray energy range benefit from enhanced photoelectron escape depth and thus bulk sensitivity, free-electron final states and better definition of the surface-perpendicular momentum important for three-dimensional materials. The RIXS endstation includes a high-resolution spectrometer delivering *E*/Δ*E* > 11000 at 1 keV photon energy, which takes the RIXS experiment from the energy scale of charge-transfer and crystal-field excitations to that of orbital and magnetic excitations, and a rotating platform to study the dispersion of these excitations in **k**-space.

Below we describe in detail the concepts, technical realisation and performance of the ADRESS beamline, focusing mostly on the beamline optics.

## 2. Undulator source

The photon source of the ADRESS beamline is the undulator UE44 (for the 44 mm period length) installed in the medium-length straight section 3M of SLS. It has 75 periods, making a total length of 3.4 m. A preliminary account of this device is given by Schmidt *et al.* (2007 ▶) and a detailed description will be published elsewhere. Briefly, UE44 is based on the APPLEII design with permanent magnets (Sasaki *et al.*, 1993 ▶). All four arrays can be shifted in the longitudinal direction, which delivers full control of the light polarization (including circular and linear with the polarization vector variable within 0–180°). Furthermore, following the original idea by Carr (1991 ▶) (see also Lidia & Carr, 1994 ▶; Carr *et al.*, 1995 ▶), the longitudinal shifts of the four arrays can also control the energy without changing the undulator gap. Our UE44 is the first practical realisation of this fixed-gap concept.

**Figure 1:**
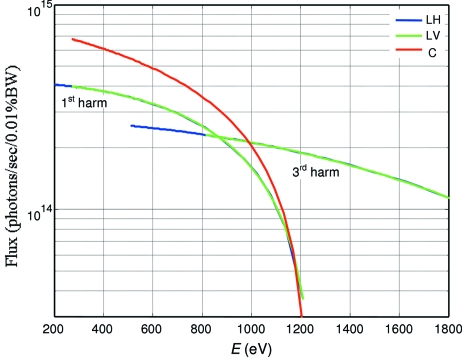
The flux produced by UE44 within the central cone for a current of 400 mA in the ring for the linear horizontal (LH), linear vertical (LV) and circular (C) polarizations. The linear polarizations follow the same flux curve but start from different energies.

Fig. 1 ▶ shows calculated flux within the central cone of radiation for various polarizations. The flux curves for all linear polarizations are identical, but they start from different energies. The source formed by UE44 is characterized by a vertical × horizontal FWHM size of *s*_V_ × *s*_H_ = 33 µm × 252 µm and angular divergence of the central cone of 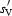 × 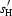 = 33 µrad × 111 µrad around 1 keV photon energy including the electron beam size and divergence. An increase of the diffraction contribution towards lower energies affects mostly 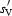 which increases to 59 µrad at 300 eV.

## 3. Beamline optics

### 3.1. Optical scheme

The beamline optics is based on the proven scheme of a plane-grating monochromator (PGM) operating in collimated light (Follath & Senf, 1997 ▶). PGMs in general (Petersen *et al.*, 1995 ▶; Peatman, 1997 ▶) benefit from high resolution, high flux owing to the absence of the entrance slit allowing the beamline to accept the whole central cone of undulator radiation, and wide energy range covered with one grating. The collimated light operation of the PGM, compared with the original SX700 scheme (Petersen, 1982 ▶) and later VLS grating schemes (Polack *et al.*, 2007 ▶), brings an additional flexibility (Follath & Senf, 1997 ▶): one can steer the PGM through different operation modes characterized by high resolution, high flux or maximal high-order suppression by tuning the cosine ratio *C*_ff_ = cosβ/cosα between the incidence and exit angles at the grating, α and β. On the practical side, our choice of optical scheme was supported by good experience with the collimated-light PGMs at BESSY as well as with two other beamlines of this type operating at SLS (Flechsig *et al.*, 2001 ▶; Quitmann *et al.*, 2001 ▶).

**Figure 2:**
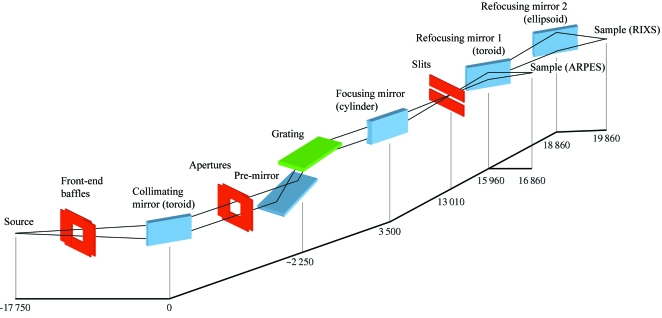
Optical layout of the beamline. The optical scheme is the collimated-light PGM. The dimensions are in mm.

The optical layout of the ADRESS beamline is shown in Fig. 2 ▶. The source is the UE44 undulator. The front-end baffles select the central cone of radiation. The collimating mirror (CM) is a toroid which converts the divergent beam from the undulator source into a beam which is parallel in the vertical (dispersive) plane. The monochromator situated downstream consists of a plane pre-mirror (PM) and selectable gratings, dispersing the beam in photon energies. Three gratings with constant groove densities *N* of 800, 2000 and 4200 lines/mm are used to provide even coverage of the beamline resolution and transmission parameters (see below). Downstream from the monochromator is a focusing mirror (FM). It is a cylinder which focuses the dispersed collimated beam onto the exit slit, producing monochromatic light. In the horizontal (non-dispersive) plane, the FM has no focusing properties and the beam from the undulator is directly focused by the CM onto the exit slit, producing a stigmatic focus. Owing to radiation safety the optical elements up to FM are enclosed in a hutch with lead walls. The first refocusing mirror (RM1) is a toroid which refocuses the beam onto the sample in the first (ARPES) endstation. When the RM1 is taken away from the beam path, the second refocusing mirror (RM2) having an ellipsoidal shape refocuses the beam onto the second (RIXS) endstation. Parameters of all optical elements are summarized in Table 1 ▶.

It should be noted that an alternative horizontal focusing scheme is possible where the CM is a cylinder and the focusing is performed by a toroidal FM. Owing to larger demagnification this scheme allows reduction of the horizontal spot size. However, this is associated with an increase in the horizontal beam divergence at the exit slit, in our case ∼40%. The corresponding increase of the light footprints at the refocusing optics is prohibitive for the ellipsoidal RM2 because of technological difficulties in manufacturing large high-quality ellipsoidal surfaces. Moreover, this scheme makes the resolution slightly worse (see below). Other horizontal focusing schemes involving intermediate collimation between toroidal CM and FM similarly to the vertical focusing (Flechsig *et al.*, 2001 ▶; Quitman *et al.*, 2001 ▶) are less practical because of additional coupling between the two mirrors which complicates the beamline alignment.

### 3.2. Resolution optimization

The parameters and actual positions of the optical elements in Fig. 2 ▶ were optimized in pursuit of high resolving power *E*/Δ*E* > 30000 at 1 keV photon energy. Ray-tracing calculations were performed using the code *PHASE* (Bahrdt *et al.*, 1995 ▶). The source was taken to have a vertical × horizontal FWHM size of 33 µm × 252 µm and divergence 33 µrad × 111 µrad (see above; the increase of vertical divergence of our source at low energies had an insignificant effect on the beamline resolution). The slope errors (SEs) of the optical elements were the measured values from Table 1 ▶.

The beamline resolution Δ*E* has the following contributions.

(i) The spot-size-limited resolution Δ*E*_spot_ determined by imaging of the photon source onto the exit slit in the presence of aberrations and slope errors of all optical elements. This contribution is determined as Δ*E*_spot_ = *S*_V_ *E* ^2^cosβ/(1.24*kNf*), where *S*_V_ is the focused vertical FWHM spot size in µm normally found from numerical ray-tracing calculations including the slope errors, the photon energy *E* is in eV, β is the exit angle at the grating, *k* is the diffraction order, and *f* is the exit arm (formula adopted from West & Padmore, 1987 ▶).

(ii) Diffraction-limited resolution Δ*E*_diff_ owing to the diffraction limit on the grating. Normally minor compared with Δ*E*_spot_, this contribution is determined by the expression *E*/Δ*E*_diff_ = *Nl* (Follath *et al.*, 1998 ▶) which represents the number of grooves within the length *l* (restricted by the optical surface) illuminated by the central cone of the undulator radiation (coherent in the vertical plane because the photon beam emittance in this plane is diffraction limited with a negligible contribution of the electron beam emittance).

(iii) Real operation of the beamline with non-zero slit introduces a contribution of the exit slit limited resolution Δ*E*_slit_, which is determined analytically by a formula identical to that for Δ*E*_spot_ with *S*_V_ replaced by the slit width *d* (West & Padmore, 1987 ▶).

The total resolution Δ*E* is the geometrical sum of the three contributions, Δ*E* = (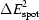 + 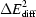 + 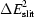)^1/2^. Conventionally, our further resolution analysis takes into account only Δ*E*_spot_ and Δ*E*_diff_, reflecting therefore the ultimate beamline resolution in the limit of zero exit slit opening.

Our ray-tracing analysis has suggested the following measures to reach the requested resolution:

(i) For the collimated-light PGMs the resolution is limited mainly by SEs of optical elements rather than aberrations. To identify the most critical ones, we first performed reference ray-tracing calculations for an energy of 1 keV, grating of 4200 lines/mm and *C*_ff_ = 10 with typical manufacturing SE values, which are around 0.1/0.5 arcsec in the meridional/sagittal directions for the plane optics and 0.5/2.5 arcsec for the toroidal optics. These calculations returned *E*/Δ*E* ≃ 20100. Then we checked the increments of *E*/Δ*E* upon setting each SE to zero. As already known for the PGMs, the most critical was the meridional SE of the grating, which showed a jump of *E*/Δ*E* by ∼26400 relative to the reference value. This SE had to be specified below 0.07 arcsec, close to the present technological limit, in order to reach the requested beamline resolution. The second critical was the sagittal SE of the FM, where the increase was ∼1700. It was specified to be below a more relaxed value of 1.5 arcsec. Other SEs had insignificant effect on the resolution.

(ii) The focus on the exit slit was made stigmatic in order to minimize the influence of the grating and exit slit misalignments on resolution.

(iii) The horizontal focusing is performed by the toroidal CM without any effect of the cylindrical FM. Our ray-tracing analysis has shown that this improves maximal *E*/Δ*E* by ∼1000 compared with the alternative scheme where the horizontal focusing is performed by a toroidal FM (see above).

### 3.3. Resolution parameters

**Figure 3:**
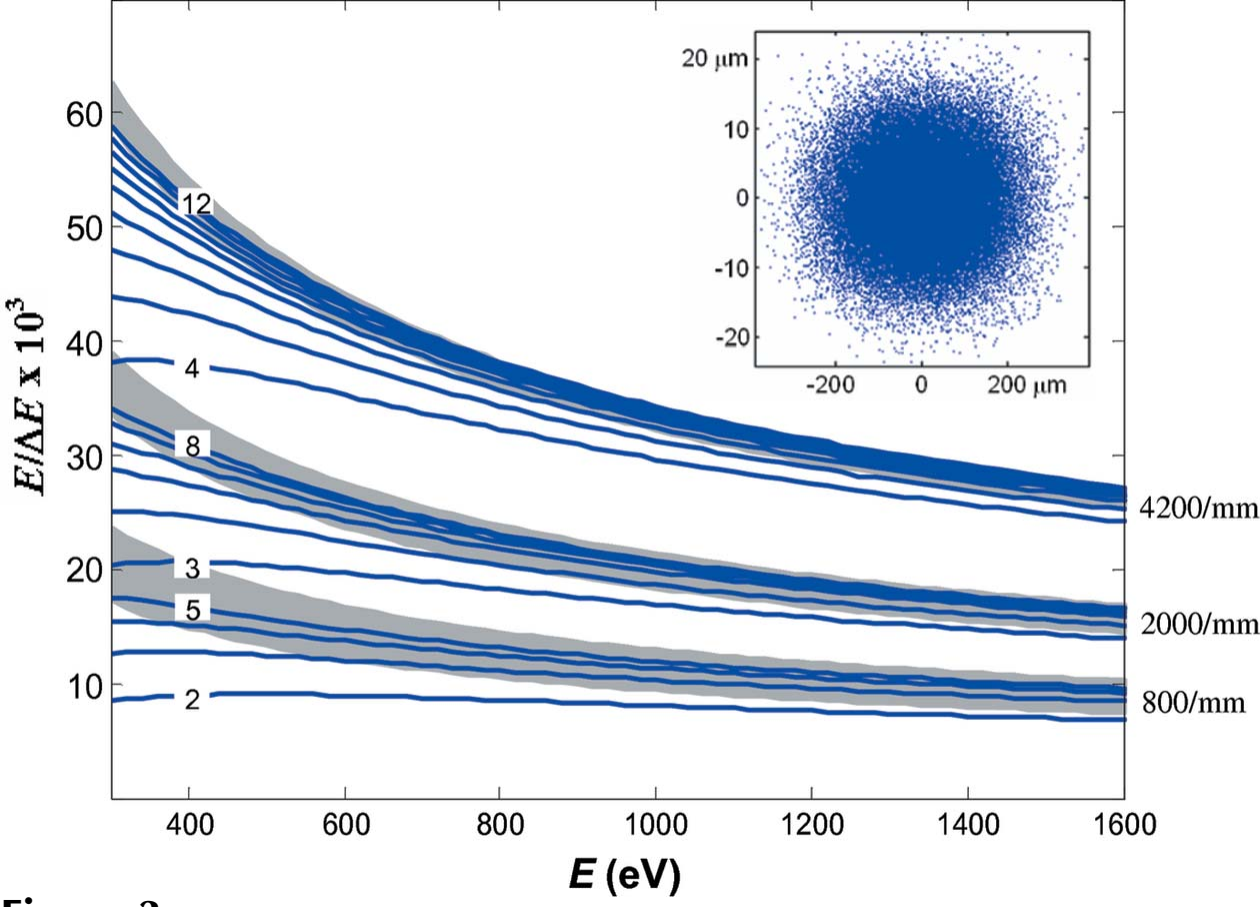
Theoretical *E*/Δ*E*, including the spot size and diffraction contribution, as a function of photon energy. The three bunches of lines correspond to the three gratings (indicated on the right) and the individual lines within each bunch to variation of *C*_ff_ by integer numbers (within the limits indicated on the left). The shaded bands display *E*/Δ*E* without the diffraction contribution, with their edges corresponding to the *C*_ff_ limits of the bunches. The insert shows a typical spot at the exit slit.

Results of the resolution calculations with the optimized beamline geometry are shown in Fig. 3 ▶. First, the insert displays a typical spot at the exit slit generated by ray-tracing calculations with the 4200 lines/mm grating and *C*_ff_ = 4. The vertical × horizontal FWHM spot size is *S*_V_ × *S*_H_ = 14.1 µm × 228 µm. Its theoretical limit is set by direct demagnification of the undulator source by the ideal beamline with zero SEs and aberrations. With our optical scheme the demagnification in the vertical directions is given by *r*′/(*rC*_ff_), where *r* and *r*′ are the source-to-CM and FM-to-slit distances, respectively, and in the horizontal direction by *r*′′/*r*, where *r*′′ is the CM-to-slit distance. This sets the theoretical limit at *S*_V_ × *S*_H_ = 4.4 µm × 185 µm. The additional broadening for the real beamline is due mostly to the SEs (predominantly the meridional SE of the grating, see above) rather than aberrations, which is reflected by the Gaussian profile of the spot.

The plots in Fig. 3 ▶ show the calculated Δ*E* (including the Δ*E*_spot_ and Δ*E*_diff_ contributions) represented as *E*/Δ*E* depending on photon energy. The three bunches of lines correspond to the three gratings, and variation of *E*/Δ*E* through each bunch to variation of *C*_ff_ by integer values between the indicated limits. The low limit is chosen here as the floor function of the flux-optimal *C*_ff_ (delivering maximal overall flux in the region 700–1200 eV, see below) and the high limit as the maximal integer *C*_ff_ for which the light footprint on the grating does not split beyond its optical surface of 90 mm through the whole shown energy range. The increase of *C*_ff_ results in improvement of resolution owing to increasing demagnification of the photon source. The plot shows that for the 4200 lines/mm grating and *C*_ff_ = 10 the theoretical *E*/Δ*E* is about 33700 at 1 keV photon energy. The shaded bands in the plot display *E*/Δ*E* determined by Δ*E*_spot_ without the diffraction contribution Δ*E*_diff_. The edges of the bands correspond to the *C*_ff_ limits of the above bunches. Obviously, the diffraction contribution becomes significant at low energies and small *C*_ff_ values when the incidence angle on the grating becomes less grazing.

The theoretical resolution was verified by characteristic X-ray absorption spectra of a few gases. The measurements were performed in a gas cell with gas pressure around 1 × 10^−2^ mbar. The monochromator was set to *C*_ff_ = 2.15 and the exit slit to 10 µm.

**Figure 4:**
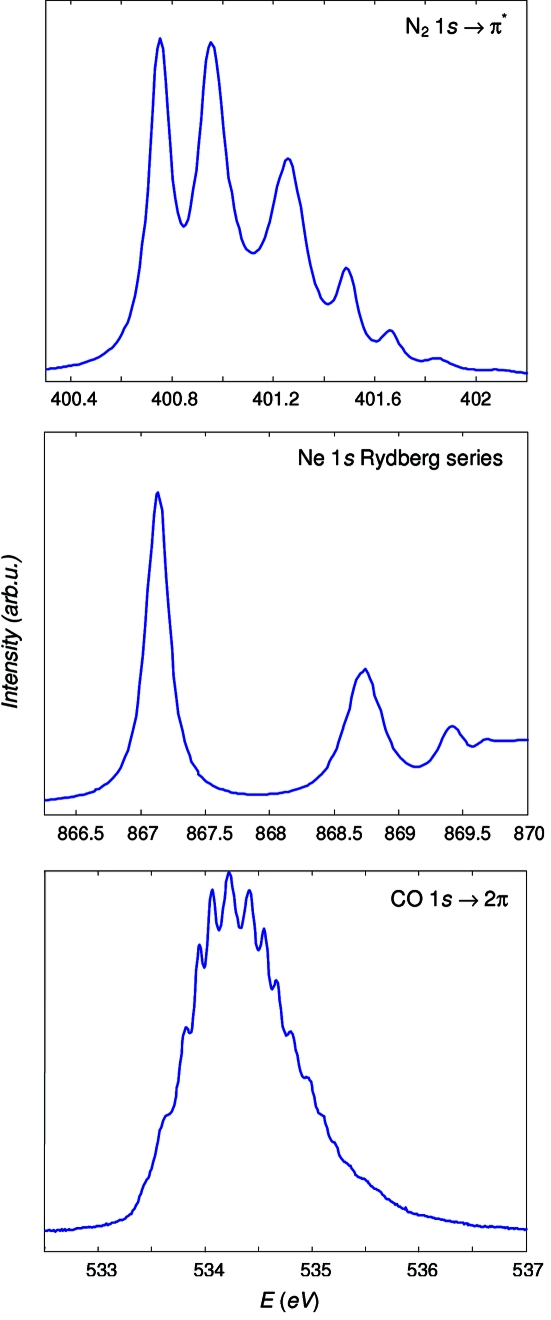
A few experimental X-ray absorption spectra of gases characteristic of energy resolution: N 1*s* → π^*^ resonance of N_2_ (800 lines/mm grating), 1*s* Rydberg series of Ne (800 lines/mm) and O 1*s* → 2π resonance of CO (4200 lines/mm).

Fig. 4(*a*) ▶ shows a typical absorption spectrum of N_2_ at the 1*s* → π^*^ resonance. Its fine structure is due to vibrational levels of the π^*^ state. The spectrum was measured with the 800 lines/mm grating. We have estimated Δ*E* (now including the Δ*E*_slit_ contribution) by comparing the experimental ratio of the first minimum to the third maximum (Chen & Sette, 1989 ▶) with that in a simulated spectrum (available online at http://slsbl.web.psi.ch/beamlines/nitrogen-resolution.shtml). A Lorentzian width of 112 meV was assumed, which is the lower limit of the diverging data found in the literature. Such a conservative estimate demonstrated *E*/Δ*E* at least better than 8500 which is consistent with the theoretical *E*/Δ*E* of ∼9600 for these beamline settings (including *E*/Δ*E*_slit_ ≃ 31000). This figure is close to the limit of resolution sensitivity of the N_2_ spectra, which is limited by some uncertainty of the Lorentzian width as well as by noise in the experimental spectra. We could hardly identify any resolution improvement with higher-*N* gratings.

Fig. 4(*b*) ▶ shows a spectrum of Ne at the 1*s* Rydberg series measured with the same 800 lines/mm grating. To estimate the resolution, we fitted the first spectral peak with a Voigt profile having a Lorentzian width of 252 meV as reported by Kato *et al.* (2007 ▶). This yielded *E*/Δ*E* ≃ 8000 which again conforms well to the theoretical *E*/Δ*E* for these beamline settings of about 8400 (including *E*/Δ*E*_slit_ ≃ 20000).

Fig. 4(*c*) ▶ shows an O 1*s* absorption spectrum of CO. Its first peak is the O 1*s* → 2π resonance with fine structure owing to the vibrational levels in the 2π state. The spectrum was measured with the 4200 lines/mm grating. To our knowledge, this is the best resolved spectrum of CO published to date. Fitting the spectrum with a sequence of Voigt profiles with a Lorentzian width of 156 meV (Tanaka *et al.*, 2005 ▶) yielded a *E*/Δ*E* value of at least better than 15000. This figure is at the resolution sensitivity of the CO spectrum, whereas the theoretical *E*/Δ*E* for these beamline settings is about 36400.

Further evidence of the beamline resolution was found from the energy width of the elastic line in the RIXS spectra (see below). With the 800 lines/mm grating, *C*_ff_ = 2.15 and an exit slit of 10 µm, we could achieve a linewidth at the Cu *L*_3_ edge (930 eV) of 120 meV (Schlappa *et al.*, 2009 ▶) corresponding to a combined *E*/Δ*E* of 7750. If we accept a theoretical *E*/Δ*E* of our RIXS spectrometer of 12000 at this energy (Ghiringhelli *et al.*, 2006 ▶), the beamline contribution to the linewidth appears better than *E*/Δ*E* = 10000, overshooting the theoretical value of 7950 for these beamline settings.

Although the above experimental X-ray absorption or RIXS spectra do not allow us to verify the ultimate *E*/Δ*E* for the 2000 and 4200 lines/mm gratings, the resolution achieved with the 800 lines/mm grating confirms the theoretical focused spot size *S*_V_. This suggests that *E*/Δ*E* will proportionally increase with the groove density towards the ultimate design figures. With the ARPES endstation coming into operation we will be able to reliably control the ultimate resolutions by measurement of photoemission spectra at the Fermi edge.

Practically, we have found that the beamline resolution can be severely affected by vibrations coming with the turbulent flow of cooling water through the gratings and especially the PM. Cooling of the sagittally mounted CM has only a negligible effect on the resolution, but causes notable vibrations of the horizontal beam position. It is therefore crucial to keep the water flow through all these optical elements at the minimal necessary level. Apart from the usual manual adjustment for each element, we remotely control the main pump of the cooling system to adjust the total water flow according to the *K*-value of the undulator and thus total heat load on the beamline.

### 3.4. Transmission optimization

As both RIXS and soft X-ray ARPES experiments are characterized by extremely low signal, the flux performance of the beamline is of paramount importance. To achieve the best beamline transmission, a few measures have been taken: (i) glancing incidence angles at all mirrors (see Table 1 ▶); (ii) gratings were chosen with the minimal *N* necessary to achieve the requested resolutions balanced with the endstation resolutions; (iii) optimization of the grating profile parameters.

Profiles of the gratings were optimized using calculations of their efficiency with the code *REFLEC* [Schaefers *et al.* (2002 ▶); based on methods described by Nevière *et al.* (1978 ▶)]. First, we had to determine whether the gratings should be lamellar or blazed.

We compared the two options for gratings of 800 and 2000 lines/mm. The calculations were performed in the first (internal) diffraction order for the ideal grating profiles with an apex angle of 90°, and realistic profiles with apex angles of around 170° for the blazed gratings and 165° for the lamellar ones (data by ZEISS). The profile parameters were optimized for an energy of 930 eV and a *C*_ff_ of 2.25: the blaze angle ϕ_blaze_ was 0.8° for the 800 lines/mm grating and 1.3° for the 2000 lines/mm grating; for the lamellar option the groove depth *h* and duty cycle *c*/*d* (at the groove half-height for the realistic profiles) were 11.0 nm and 0.69, respectively, for the 800 lines/mm grating, and 5.5 nm and 0.6 for the 2000 lines/mm grating (the procedure to determine *h* and *c*/*d* is described below). An r.m.s. surface roughness of 0.5 nm was assumed in all cases.

**Figure 5:**
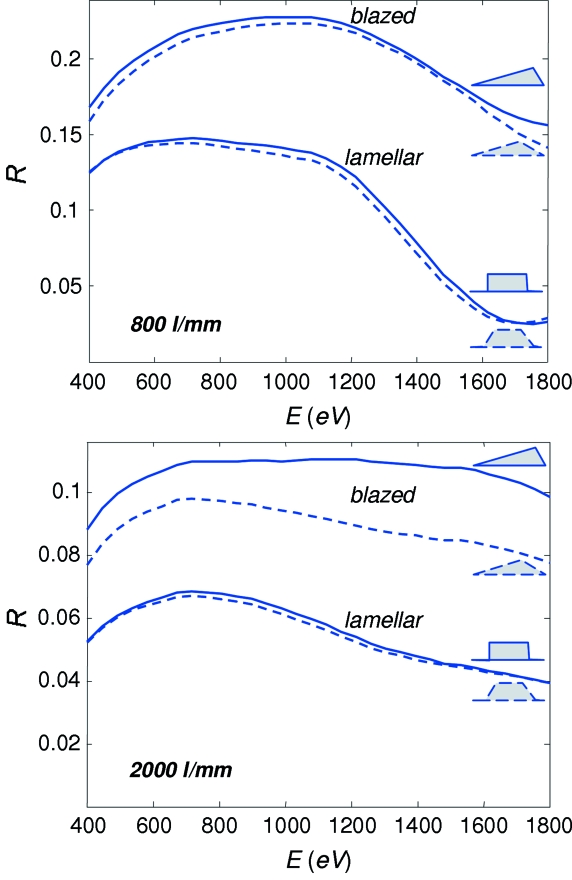
Calculated efficiency of blazed and lamellar gratings in the first diffraction order with *N* = 800 lines/mm and 2000 lines/mm in the case of an ideal 90° apex angle (solid lines) and a realistic obtuse one (dashed lines) as shown on the right. The profile parameters are optimized for the energy 930 eV and *C*_ff_ = 2.25. The blazing has a larger effect at smaller *N*.

Fig. 5 ▶ shows the calculated energy dependences of efficiency *R*(*E*). The blazed gratings demonstrate remarkable flatness of *R*(*E*) which is, surprisingly, even superior to their lamellar counterparts. For the 800 lines/mm case, the blazed profile has a higher *R*(*E*) compared with the lamellar through the whole energy region from 300 to 1800 eV. In the case of ideal grating profiles (solid lines) the increase is on average about a factor of two. Introduction of the realistic profiles (dashed lines) does not change *R*(*E*) much for both gratings, and the blazed grating retains an immense advantage. Owing to this analysis for the 800 lines/mm grating we have opted for the blazed profile with an aim to deliver maximal flux at moderate resolution. The fact that the blazed gratings suffer from a poorer higher-order suppression (HIOS) compared with the lamellar grating was less important for our beamline operating at rather high energies. For the 2000 lines/mm grating, Fig. 5 ▶ shows that in the ideal case the increase of *R*(*E*) owing to the blazed profile is again about a factor of two. However, when the realistic profiles are introduced, the blazed profile shows much stronger degradation of *R*(*E*) (physically the degradation occurs owing to a decrease of the reflecting facets area with an increase of the apex angle and *N*) compared with its lamellar counterpart, reducing the advantage to a factor of about 1.5. We considered this less significant in view of a more complicated manufacturing process and thus higher prices and potential quality problems for the blazed gratings. For the 2000 lines/mm and, similarly, 4200 lines/mm gratings we have therefore opted for the lamellar profile.

The second step was to optimize the *h* and *c*/*d* parameters of the lamellar gratings. Commonly the optimization is performed by the manufacturer for a requested energy and *C*_ff_ value. However, the latter is *a priori* unknown, because its flux optimal value significantly depends on energy, *N* and profile parameters of the grating [the traditional *C*_ff_ = 2.25 goes back to the original SX-700 monochromator (Petersen, 1982 ▶) where this value was optimized for a particular energy range around 700 eV and a grating of 1200 lines/mm]. The optimal *C*_ff_ should therefore be determined in an optimization procedure simultaneously with *h* and *c*/*d*. Furthermore, the variation of *C*_ff_ implies variation of the deviation angle at the grating and thus at the PM, with consequent variation of reflectivity at this optical element. Therefore, the target function in the optimization procedure should be the total transmission *R*_2_ = *R*_PM_ *R*_grating_ of the PM and grating pair.

**Figure 6:**
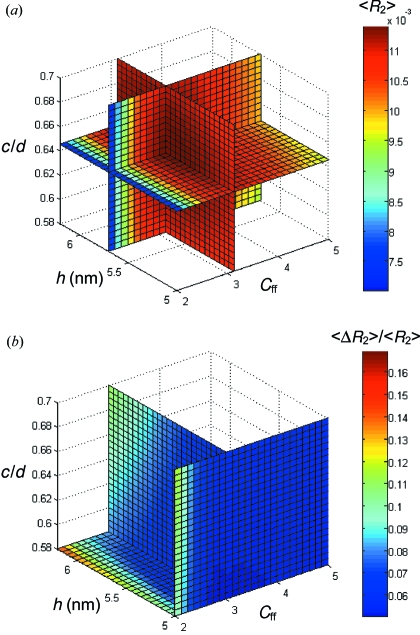
Optimization of the grating parameters for the 2000 lines/mm grating in the first diffraction order. Transmission of the PM and grating pair *R*_2_ over the energy interval 700–1200 eV, average 〈*R*_2_〉 (*a*) and relative variation 〈Δ*R*_2_〉/〈*R*_2_〉 (*b*). The crossing planes show the position of their maximum and minimum, respectively. The optimal values of *h* and *c*/*d* are chosen slightly shifted from the exact transmission maximum to improve the flatness of the energy dependence.

The optimization used the transmission 〈*R*_2_〉 in the first (internal) diffraction order averaged over eight points in an energy interval from 700 to 1200 eV. The apex angle was 164° for *N* = 2000 lines/mm and 163° for *N* = 4200 lines/mm (data by ZEISS), and r.m.s. roughness was 0.5 nm. Fig. 6(*a*) ▶ shows 〈*R*_2_〉 calculated for the 2000 lines/mm grating over a three-dimensional grid of *h*, *c*/*d* (in the following, *c* refers to the top of the grooves, which is standard for the input of *REFLEC*) and *C*_ff_. The three crossing planes identify the broad transmission maximum centered at *h* = 5.7 nm, *c*/*d* = 0.645 and *C*_ff_ = 3.125. Fig. 6(*b*) ▶ shows an additional optimization criterion, flatness of the 〈*R*_2_〉 energy dependence characterized by relative r.m.s. deviation 〈Δ*R*_2_〉/〈*R*_2_〉 calculated over the above eight points. Obviously, it gradually improves with decrease of *h* and *c*/*d* and reaches its minimum at their smallest values. We have therefore chosen the optimal *h* and *c*/*d* values slightly smaller than in the *R*_2_ maximum, which notably improved the flatness at insignificant (<0.5%) degradation of *R*_2_. However, the minimum of 〈Δ*R*_2_〉/〈*R*_2_〉 appears at almost the same *C*_ff_ value as the 〈*R*_2_〉 maximum. This analysis yielded the flux-optimal parameters for the 2000 lines/mm grating as *h* = 5.5 nm, *c*/*d* = 0.63 and *C*_ff_ = 3.125. The same optimization for the 4200 lines/mm grating has yielded the *R*_2_ maximum at *h* = 4.75 nm, *c*/*d* = 0.65 and *C*_ff_ = 4.75. To improve the flatness, the flux-optimal parameters for this grating were chosen as *h* = 4.5 nm, *c*/*d* = 0.64 and *C*_ff_ = 4.75. As mentioned above, optimization of the gratings on HIOS was not a concern for our beamline.

We have also determined the flux-optimal *C*_ff_ for the 800 lines/mm blazed grating by optimization of 〈*R*_2_〉 on a one-dimensional grid of *C*_ff_ with the blaze angle varying simultaneously with *C*_ff_ according to ϕ_blaze_ = (α + β)/2. The maximum was found at *C*_ff_ = 2.15 and ϕ_blaze_ = 0.8°.

### 3.5. Flux parameters

**Figure 7:**
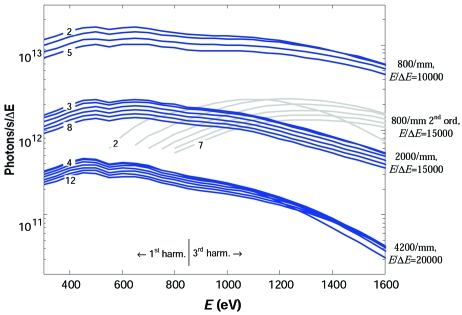
Theoretical flux at the sample as a function of photon energy for the LH polarized light and 400 mA current in the ring. The three bunches of solid lines correspond to the three gratings (indicated on the right) and the bunch shown shaded to the blazed 800 lines/mm grating operating in the second order. Variations of flux from the bottom to top edges of each bunch correspond to variation of *C*_ff_ in integer values within the limits indicated on the left. The flux is normalized to the bandwidth corresponding to the *E*/Δ*E* values typical of each grating (indicated on the right).

Fig. 7 ▶ shows the theoretical flux that the beamline delivers at the sample (after the RM) as a function of photon energy for a current in the ring of 400 mA and LH polarized light, with a crossover between the first and third harmonic radiation at around 865 eV (Fig. 1 ▶). Similarly to the resolution data in Fig. 3 ▶, the three bunches of solid lines correspond to the three gratings. Variation of flux from the bottom to top edges of each bunch corresponds to variation of *C*_ff_ in integer values, the same as in Fig. 3 ▶ (from the floor function of the flux-optimal *C*_ff_ in the 700–1200 eV region to the maximal integer *C*_ff_ for which the light footprint stays within the optical surface of the gratings). The flux is normalized to the energy-dependent bandwidth corresponding to the *E*/Δ*E* values typical of each grating. At 1 keV photon energy and flux-optimal *C*_ff_ value, the theoretical flux for the 800 lines/mm grating  is 1.3 × 10^13^ photons s^−1^ in a bandwidth corresponding to *E*/Δ*E* = 10000, for the 2000 lines/mm grating it is 1.7 × 10^12^ photons s^−1^ corresponding to *E*/Δ*E* = 15000, and for the 4200 lines/mm grating it is 2.4 × 10^11^ photons s^−1^ corresponding to *E*/Δ*E* = 20000.

Another bunch in Fig. 7 ▶ (shown in grey) represents the same flux curves calculated for the 800 lines/mm grating operating in the second diffraction order. Compared with the 2000 lines/mm grating in the first order, this operation mode, having close resolution parameters, delivers higher flux in the high-energy region above 1 keV. Such a high second-order intensity is due to the blazed profile of the 800 lines/mm grating.

**Figure 8:**
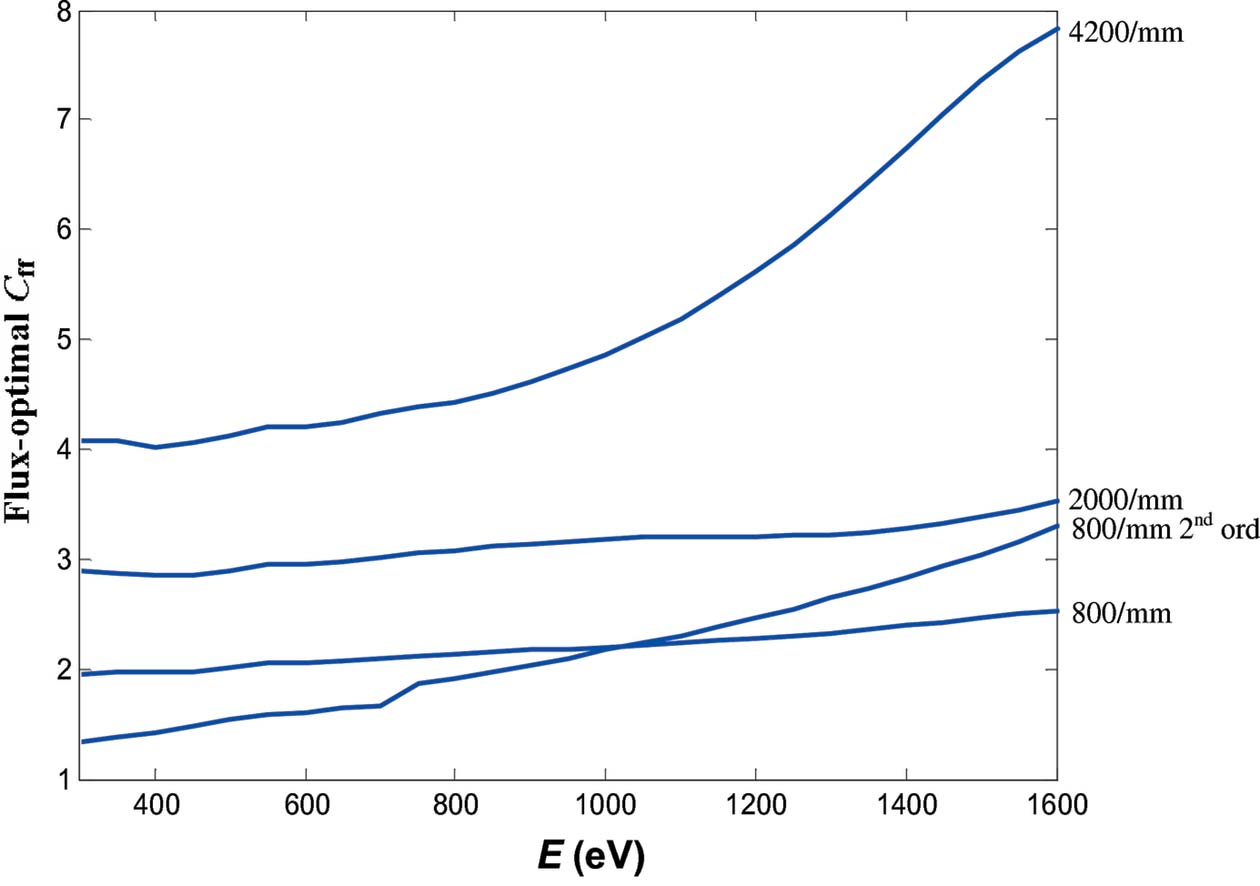
Calculated energy dependences of the flux-optimal *C*_ff_.

A closer look at the flux curves in Fig. 7 ▶ shows that the flux-optimal *C*_ff_ value increases with energy and groove density. This is illustrated by calculated energy dependences of the flux-optimal *C*_ff_ in Fig. 8 ▶. The energy variation is particularly large for the 800 lines/mm grating in the second order and 4200 lines/mm grating.

**Figure 9:**
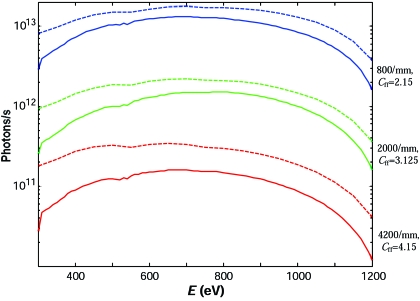
Experimental flux curves (solid lines) measured on the photodiode with 400 mA current in the ring, first harmonic of the LH polarized light and 20 µm exit slit, for the three gratings with flux-optimal *C*_ff_ values (indicated on the right). The corresponding theoretical flux curves (dashed lines) are calculated for the same parameters. The experimental flux has potential for improvement with better alignment of the electron trajectories and UE44 magnetic lattice.

The theoretical flux was verified by measurements with a photodiode (AXUV100 from International Radiation Detectors) using the responsivity data from the manufacturer (available at http://www.ird-inc.com/). The diode was installed after the exit slit (before the refocusing optics). The beamline was operated with the nominal current in the ring of 400 mA, the first harmonic of the LH radiation, the flux-optimal *C*_ff_ values for each grating, and the exit slit fixed to 20 µm (*E*/Δ*E* varying with energy). The measurements were performed after three years of regular beamline operation. The experimental flux curves are shown in Fig. 9 ▶ (solid lines) in comparison with theoretical ones (dashed lines) calculated with the same beamline set-up. Certain reduction of the experimental flux can be attributed, apart from imperfections of the optical elements and their accumulating contaminations, mainly to remnant misalignments of the electron trajectories and UE44 magnetic lattice (in fact, we could reach the experimental width of the undulator radiation peaks corresponding to some 55 periods rather than the physical 75) which can be improved in future. The larger flux reduction seen for the 4200 lines/mm grating, going along with about 70% inhomogeneity of efficiency observed along the optical surface, can be expected for a grating with a groove density close to the present technological limit. Our experimental flux performance can be compared with that of one of the worldwide best high-resolution soft X-ray beamlines, BL25SU at SPring-8 (Saitoh *et al.*, 2000 ▶). For the same bandwidth corresponding to *E*/Δ*E* = 10000 (with the 800 lines/mm grating for ADRESS and 600 lines/mm for BL25SU) the flux delivered by the ADRESS beamline is better overall by a factor above 90, and for *E*/Δ*E* = 15000 (the 2000 lines/mm and 1000 lines/mm gratings, respectively) above 8.

The excellent flux performance of the beamline has resulted from a few factors: the energy of the ring optimized for the soft X-ray photon energy range, an optical scheme of the PGM without entrance slit allowing acceptance of the whole central cone of undulator radiation, and all above measures to optimize the beamline transmission.

### 3.6. Refocusing optics

Refocusing was particularly demanding for the RIXS endstation because slitless operation of our high-resolution RIXS spectrometer (see below) required squeezing the vertical spot size to below 5 µm FWHM. In order to reduce flux loss, we have restricted ourselves to one-mirror refocusing systems and small grazing incidence angle α = 1°. We have investigated two options, a toroidal refocusing mirror (T-RM) and an ellipsoidal refocusing mirror (E-RM). It should be noted that the ellipsoidal refocusing optics can only be used if the beamline focus is stigmatic.

Model ray-tracing calculations were performed with the source taken as the typical spot produced by the beamline at the exit slit (see above) which has dimensions *S*_V_ × *S*_H_ = 14.1 µm × 228 µm (such a source is roughly equivalent to the exit slit open to a width of *S*_V_). The source-to-sample distance was taken as 7000 mm, and the meridional/sagittal r.m.s. SEs as their typical values of 0.5/1.5 arcsec for the T-RM and three times those for the E-RM. The spot size at the sample, *w*, resulting from the ray-tracing calculations has in fact three contributions: nominal demagnification of *S*_V,H_ by the ratio *r*/*r*′ of the object and image distances as *S*_V,H_ *r*/*r*′, SEs of the mirror 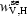 and aberrations 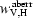 for both vertical and horizontal directions. The contributions add up geometrically as *w*_V,H_ = [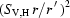 + 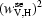 + 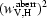]^1/2^. 

**Figure 10:**
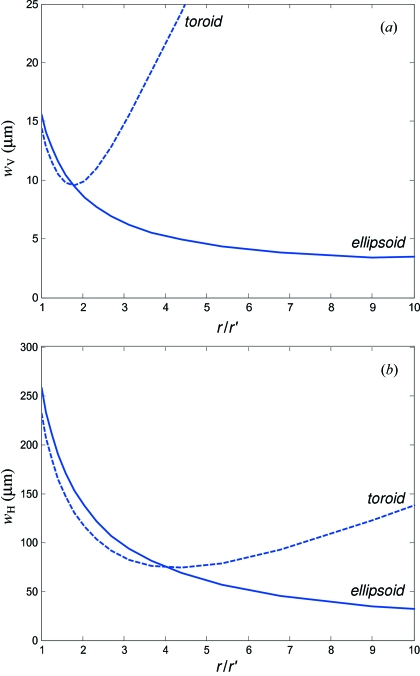
Theoretical vertical (*a*) and horizontal (*b*) FWHM spot size at the sample as a function of the nominal demagnification *r*/*r*′ for refocusing with a typical toroidal and ellipsoidal mirror. Owing to reduced aberrations the latter allows much smaller spot size when increasing *r*/*r*′.

Fig. 10(*a*) ▶ shows dependences of the vertical spot size *w*_V_ on the nominal demagnification *r*/*r*′ calculated by ray-tracing for the T-RM in comparison with the E-RM. In the region of small *r*/*r*′, the former outperforms the latter because of smaller SEs. With increase of *r*/*r*′, however, *w*_V_ produced by the T-RM rapidly reaches its minimum of ∼10 µm at *r*/*r*′ ≃ 1.8, where the growing 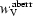 contribution starts to prevail over the decreasing 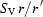 one, and the spot starts to blur (see also Peatman, 1997 ▶). The situation is different for the E-RM, which is in principle the ideal point-to-point focuser: owing to smaller aberrations, *w*_V_ carries on decreasing towards much larger *r*/*r*′ and reaches its minimum of ∼3.4 µm at *r*/*r*′ ≃ 9. The use of the E-RM thus allows more effective demagnification at large *r*/*r*′.

Fig. 10(*b*) ▶ shows the same comparison for the horizontal spot size *w*_H_. This case is less critical on aberrations because, owing to rather large *S*_H_, the dominating contribution in *w*_H_ is 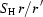 which drives it to decrease towards higher *r*/*r*′. The minimum of *w*_H_ is found at *r*/*r*′ ≃ 4 for the T-RM and well above 10 for the E-RM.

In our beamline the RIXS endstation takes the furthest position from the exit slit, which allows an *r*/*r*′ of up to 5.85 (see the optical scheme in Fig. 2 ▶). A value of *w*_V_ as small as possible should be achieved for the slitless spectrometer operation. We have therefore opted here for refocusing with the E-RM. With the actual SEs of our E-RM (Table 1 ▶) and *r*/*r*′ = 5.85 this delivers a spot size of *w*_V_ × *w*_H_ = 3.9 µm × 52 µm for the above typical spot at the exit slit, and a minimal vertical spot size of 3.1 µm in the limit of zero exit slit opening. We have verified the spot size by measurements with the RIXS spectrometer (see below) which in the zero-order diffraction mode allows direct imaging of the spot on the CCD camera. With an exit slit opening of 10 µm, the spot size was at least below 4.7 µm, which is consistent with the theoretical value within the accuracy of these measurements.

Requirements of the ARPES endstation on the spot size are mainly due to the angular resolution of the ARPES spectrometer. In our case the ultimate resolution is achieved with a spot size of less than 100 µm in both directions. With *S*_H_ being much larger than *S*_V_, the horizontal demagnification becomes more critical than the vertical one. Furthermore, the ARPES station is placed first from the slit, which restricts us to a relatively small *r*/*r*′ of up to 3.28. Owing to these two considerations a satisfactory performance can be achieved here with the simpler T-RM option, which for *r*/*r*′ = 3.28 still shows improvement of *s*_H_ without much degradation of *s*_V_. With the actual SEs of the T-RM (Table 1 ▶) the spot size on the sample for the above typical spot at the exit slit was calculated as *w*_V_ × *w*_H_ = 10.0 µm × 73.6 µm. Anyway, based on our experience with the E-RM for the RIXS endstation and in view of the importance of further reduction of *w*_H_ for experiments on mosaic samples, we plan in future to increase *r*/*r*′ and upgrade to the ellipsoidal mirror.

On the practical side, it has been crucial that the manufacturer was able to produce the E-RM with extremely small SEs (see Table 1 ▶). Furthermore, it should be noted that the ellipsoidal optics is by far more sensitive to alignment compared with the toroidal optics. We have therefore mounted the refocusing mirrors on advanced hexapod mechanics from Oxford-FMB (product information available at http://www.fmb-oxford.com/product.php?product=53) delivering three translational and three rotational fully decoupled degrees of freedom (DOFs) with a practical accuracy better than 1 µm and 1 mrad, respectively.

### 3.7. Diagnostics

The beamline is equipped with a whole bundle of standard diagnostics tools such as X-ray beam-position monitors (XBPMs) in the front end, retractable fluorescent screens along the beam path (downstream from the FM as well as in front of both RMs), the AXUV100 photodiode after the exit slit, drain current readouts at the RMs, *etc*.

**Figure 11:**
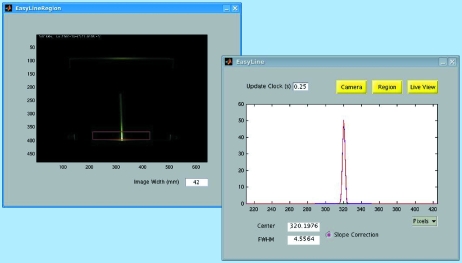
Image-processing software for evaluating the beam profile at the exit slit. Left: image with the region of interest containing the vertical line of energy-dispersed light. Right: horizontal beam profile and its Gaussian fit yielding the horizontal beam position and width.

Extremely useful for the beamline alignment and monitoring proved to be an originally developed online beam monitor installed in front of the exit slit. The monitor uses a fluorescent YAG crystal with dimensions of 24 mm × 24 mm installed slightly above the slit. The beam produces here a vertical line of energy-dispersed light. This image is streamed over the network by an IP camera with a resolution of 640 × 480 pixels (AXIS210 from AXIS Communications) and is visualized with an internet browser. A real-time image-processing software written in *Matlab* (Fig. 11 ▶) intercepts the stream, selects the region of interest around the line of light (left panel), corrects the intensity for the standard gamma-correction, compensates the line curvature caused by optical aberrations, sums up the image rows through the region to evaluate the average horizontal profile, performs a Gaussian fit of the profile to evaluate the horizontal beam position and width (right) and streams these parameters as process variables into the beamline control system. The software is available from the first author.

The above beam monitor is particularly useful with our optical scheme (Fig. 2 ▶) where the horizontal focusing at the exit slit is performed by the toroid CM decoupled from the cylinder FM. In this case monitoring of the horizontal beam width allows us to adjust the pitch angle of the CM. With the vertical curvature adopted exactly for this angle, the CM will produce vertically collimated light.

### 3.8. Optical alignment strategies

Alignment of the beamline is a complex process of optimization in a multidimensional space of the translational and angular DOFs of all optical elements. Otherwise hopeless, successful alignment requires that the optimization process is broken into well defined procedures with reduced dimensionality. As an example, we present here analysis of the vertical focusing of the beamline, directly related to the energy resolution, and demonstrate a technique to reduce this problem to fast and straightforward optimization in one dimension.

**Figure 12:**
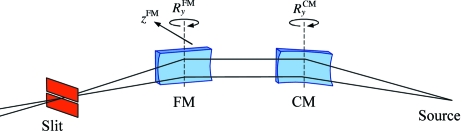
Vertical focusing scheme. The CM pitch 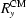, FM pitch 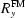 and FM transverse translation *z* ^FM^ combine in one focalization motion.

A simplified scheme of vertical focusing is given in Fig. 12 ▶. To focus the beam on the exit slit, one tweaks the FM pitch 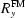. This immediately displaces the horizontal beam position from the slit center, which should be back-corrected by the FM horizontal translation *z* ^FM^. The latter, in turn, displaces the FM aperture away from the beam coming from the CM, requiring correction of the CM pitch 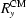. Commonly, one performs focusing by iterative tweaking of all these three motions, which is a time-consuming and less controllable procedure of optimization in three dimensions.

In fact, two conditions, (i) that the beam stays on the center of the slit and (ii) that the beam from the CM hits the center of the FM aperture, link the individual 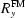, *z* ^FM^ and 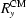 motions into one combined focalization motion (CFM) acting as one DOF. We have set up the CFM for our beamline using the following method. The *z* ^FM^ motion is chosen as a parameter of the CFM. The remaining two individual motions are taken as linear functions of *z* ^FM^ as
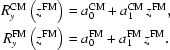
To determine the coefficients *a*, we take two arbitrary reference *z* ^FM^ points separated by a few millimeters. First, in each point we find the 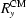 value corresponding to the center of the FM aperture. For this purpose we scan the aperture with 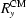 and detect the transmitted beam on the YAG crystal in front of the slit using the above beam monitor. (It may be necessary to roughly adjust 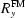 so that the whole excursion of the beam stays within the YAG crystal; the photodiode behind the slit was not suitable as a detector for this scan because its size was smaller than the excursion.) Having determined the reference 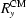 values for the two *z* ^FM^ points, we immediately find the above coefficients 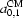 with a linear fit. Second, in each reference *z* ^FM^ point we set the determined 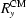 values and tweak 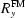 to put the beam exactly at the center of the slit. The two reference 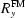 determined in this way immediately fix the coefficients 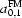. The CFM defined with this method was realised using a simple GUI-based software which upon input of the *z* ^FM^ parameter automatically calculates the corresponding 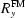 and 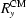, and drives the motors of all three individual motions. The accuracy of the CFM set-up can be judged by the constancy of the beam position at the slit when scanning the CFM. Normally, the deviation is less than 75 µm for the whole focalization span of *z* ^FM^.

The CFM concept allows reduction of the beamline focalization problem to a fast and unambiguous procedure of optimization in one dimension, as compared with the common iterative tweaking in three dimensions. In addition, by the very definition of the CFM, the beam always stays in the middle of the FM optical surface, which ensures the highest resolution owing to minimal SEs as well as the highest beamline transmission.

**Figure 13:**
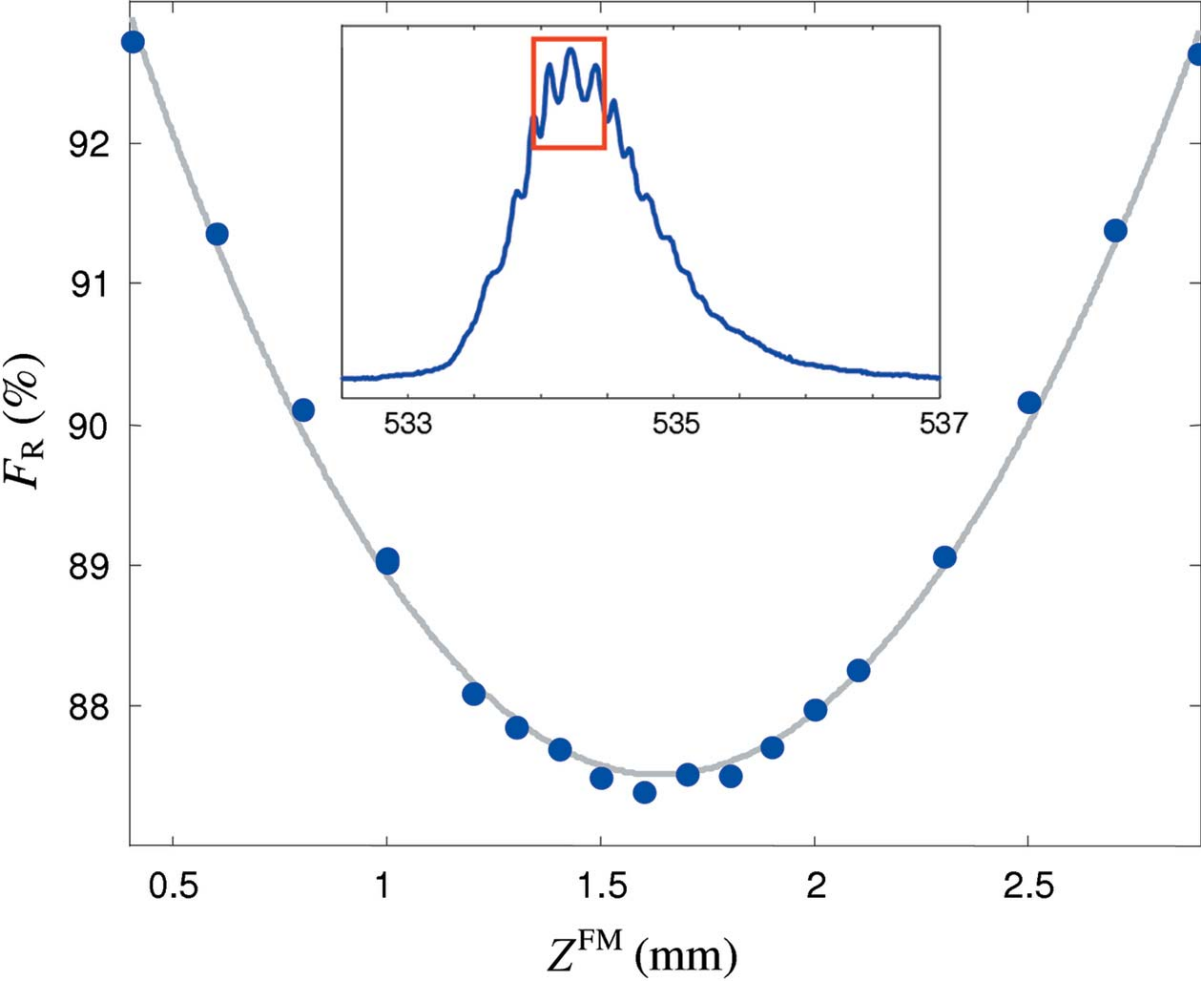
Typical focalization curve as a resolution factor *F*_R_ depending on the CFM parameter *z* ^FM^ (800 lines/mm grating, *C*_ff_ = 2.15). A cubic polynomial fit of the measured points is shown by the gray line. The insert shows the region in the O 1*s* absorption spectrum of CO used to define *F*_R_.

Fig. 13 ▶ shows typical measurements of a focalization curve as a resolution factor *F*_R_ depending on the CFM parametrized by *z* ^FM^. Here, *F*_R_ was quantified from the O 1*s* absorption spectrum of CO as the averaged minimum-to-maximum ratio of three oscillations on top of the spectrum (window in the insert of Fig. 13 ▶). A cubic polynomial fit (gray line) of the measured points puts the focal point at *z* ^FM^ = 1.64 mm. Typically, we perform full beamline focalization in 1–2 h.

## 4. Control system

**Figure 14:**
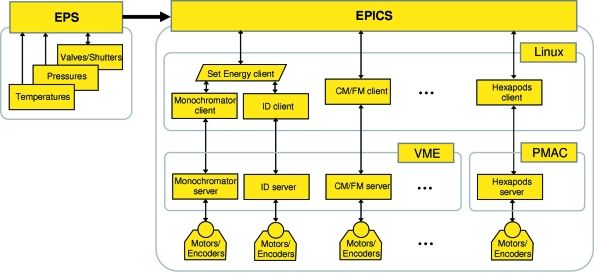
Scheme of the beamline control system.

A scheme of the beamline control system is shown in Fig. 14 ▶. It consists of two main subsystems:

(i) Equipment protection system (EPS), which surveys the temperatures and pressures interlocked to the beamline shutter, valves and water cooling. This subsystem is based on Siemens S7 programmable logic controllers (PLCs).

(ii) Beamline control based on experimental physics and industrial control system (EPICS), which is a set of open-source software tools for creating distributed real-time control systems for large-scale scientific instruments such as particle accelerators (Mooney *et al.*, 1996 ▶). On the upper level of our EPICS subsystem are client programs, which run on Linux or Windows workstations. On the lowest level are server programs. *Via* the network they communicate, on one side, with the clients using so-called channel access protocol provided by EPICS and, on the other side, with real-time computer hardware which is based on VERSAmodule Eurocard (VME) bus (IEEE 1014) and runs the Input-Output Controller (IOC) software. The VME hardware directly controls the motions of all beamline components (the insertion device, monochromator, mirrors, *etc.*) as well as analog/digital inputs and outputs. The use of the VME hardware allows real-time beamline control independent of the network latency. The two hexapod systems use a different type of real-time hardware, which is based on PMAC architecture running under Linux MicroIOC (Jansa *et al.*, 2007 ▶). The beamline motion control uses stepping motors, which are driven by units operating with the OMS58 Intelligent Motor Controller, and linear encoders from Renishaw Inc.

The EPS subsystem is incorporated into EPICS *via* mapping the PLCs to IOC channels (Staudemann, 2001 ▶). An important aspect of our control system is that these channels appear in EPICS on equal footing with other IOC channels which gives a consistent look and feel to the overall beamline instrumentation control.

## 5. Endstations

Here we briefly summarize the scientific concepts and technical implementation of our ARPES and RIXS endstations. Their detailed description will be presented elsewhere.

ARPES is a photon-in/electron-out technique which probes the electronic structure of solids in the sub-surface region. The ARPES spectra directly characterize the hole spectral function *A*(*E*,*k*) with resolution in energy and three-dimensional *k*. In the soft X-ray range an increased photoelectron escape depth results in enhanced bulk sensitivity, and free-electron final states and reduced broadening in the surface-perpendicular momentum allow reliable studies of three-dimensional systems. Excellent flux parameters of the ADRESS beamline help the notorious problem of small valence band photoexcitation cross section in the soft X-ray range.

The ARPES endstation has been available for users since autumn 2009. It features the following main instrumental concepts:

(i) Light is incident on the sample at a grazing angle of 20°, giving a gain in photoelectron yield of about a factor of two compared with the conventional 45° angle. To balance the horizontal and vertical size of the light footprint, the sample is rotated around the horizontal axis.

(ii) The manipulator (CARVING design by PSI and Amsterdam University delivering three mechanically decoupled angular DOFs) is mounted in horizontal orientation of the primary rotation axis. It is equipped with a He flow cryostat which allows working temperatures down to 10 K.

(iii) The analyser (PHOIBIOS 150 from SPECS delivering an angular resolution of about 0.07° and ultimate energy resolution better than 5 meV) is rotatable around the lens axis to change the slit orientation relative to the incoming light. When the slit is in the scattering plane, one can explore the symmetry of the valence states using the selection rules with variable polarization; when perpendicular, one can efficiently sample **k**-space with the primary manipulator rotation.

RIXS is a photon-in/photon-out bulk-sensitive technique which delivers information about the electronic structure of solids, liquids and gases resolved in atomic and orbital character (Kotani & Shin, 2001 ▶). The photon energy-loss spectra measured in RIXS reflect the spectrum of charge neutral (*e.g.* crystal field, charge transfer and spin) electronic excitations, and their photon momentum transfer dependence characterizes the dispersion of these excitations in **k**-space. This technique is particularly useful for strongly correlated systems such as transition metal oxides, rare earth systems as well as diluted systems like buried layers or nanostructures.

Our RIXS endstation has been in operation since spring 2007. It has the following features:

(i) A high-resolution spectrometer nicknamed Super Advanced X-ray Emission Spectrometer (SAXES) based on a VLS spherical grating. It delivers resolving power better than 10000 in the energy range up to 1100 eV, which for the first time takes the RIXS technique from the energy scale of charge transfer and crystal field excitations to that of orbital and magnetic excitations. The experiments are normally carried out at sample temperatures down to 10 K in order to reduce phonon broadening of the spectral structures. Design and realisation of the SAXES spectrometer have been described in detail by Ghiringhelli *et al.* (2006 ▶). A method to determine the spectrometer settings to cancel coma aberrations for any energy is described by Strocov *et al.* (2008 ▶).

(ii) A platform with five DOFs which serves as an optical bench for the spectrometer. The platform is rotatable to vary the scattering angle in six steps between 30° and 130°. This feature allows variation of photon momentum transfer and thus studies of dispersion of low-energy excitations in **k**-space.

First application examples illustrating the power of high-resolution soft X-ray RIXS in the investigation of orbital and magnetic excitations can be found by Ghiringhelli *et al.* (2009 ▶) and Schlappa *et al.* (2009 ▶).

## 6. Summary

We have presented in detail the concepts and technical realisation of the high-resolution beamline ADRESS operating in the soft X-ray energy range from 300 to 1600 eV. Below we summarize the main features of the beamline.

The undulator source is the first to fully rely on the fixed-gap concept where the gap variation functionality is abandoned, and both polarization and energy of the light are changed only by longitudinal movement of permanent magnetic arrays. The undulator gives full polarization control, delivering left- and right-circular as well as 0–180° rotatable linear polarizations.

The beamline optics adopts the scheme of PGM operating in collimated light, which allows convenient optimization of the *C*_ff_ parameter for maximal flux or resolution. The horizontal focusing on the exit slit is performed directly by the CM through the cylinder FM. The ultimate *E*/Δ*E* is above 33000 at 1 keV photon energy.

The measures to increase the beamline transmission (including glancing incidence on the mirrors, minimal grating groove densities tightly matching the required resolution, appropriate choice of the blazed *versus* lamellar gratings and with optimized profile parameters, operation at flux-optimal *C*_ff_ increasing with groove density) as well as the parameters of the ring optimized for the soft X-ray region result in experimentally verified high photon flux nearing 1 × 10^13^ photons s^−1^ (0.01% BW)^−1^ at 1 keV photon energy.

A beamline focalization method is introduced to coordinate motions of the CM and FM in one DOF and ensure fast focalization accompanied by maximal transmission. The beamline alignment was facilitated by the beam monitor allowing on-line evaluation of the horizontal beam position and profile at the exit slit. An overview of the EPICS-based beamline control system is given.

Our ARPES endstation features a grazing light incidence angle of 20°, horizontal axis of primary rotation, manipulator having three angular DOFs, and rotatable photoelectron analyzer. The RIXS endstation is equipped with the high-resolution spectrometer SAXES delivering *E*/Δ*E* ≃ 11000 at a photon energy of 1 keV. It is mounted on a rotating platform to vary the scattering angle. Ellipsoidal refocusing optics demagnifies the vertical spot size ultimately below 4 µm, allowing slitless operation of the spectrometer. Owing to low cross section of the soft X-ray ARPES and RIXS, the performance of the two endstations critically depends on the high flux delivered by the beamline.

## Figures and Tables

**Table 1:** Parameters of the optical elements The CM and PM are internally water-cooled and the gratings side-cooled; all other optical elements are not cooled. The CM was produced by ZEISS out of a substrate with cooling channels delivered by INSYNC. The PM, gratings and RMs were produced by ZEISS, and the FM by SESO.

	Shape	Grazing incidence angle (°)	Meridional/sagittal radii (mm)	Measured meridional/sagittal r.m.s. slope errors (arcsec)	Substrate/coating
CM	Toroid	1.5	573600/ 929	0.4/2.5	Si/Pt
PM	Plane	–	∞	0.052/0.094	Si/Pt
Grating, 800 lines/mm	Plane	–	∞	0.07/0.15	Si/Au
Grating, 2000 lines/mm	Plane	–	∞	0.066/0.08	Si/Au
Grating, 4200 lines/mm	Plane	–	∞	0.058/0.062	Si/Au
FM	Cylinder	1.0	∞/332	0.29/1.4	Si/Pt
RM1	Toroid	1.0	74000/24.1	0.58/0.78	Fused silica/Pt
RM2	Ellipsoid	1.0	Shape parameters: *A* = 3425 mm, *B* = *C* = 42.2 mm	1.5/3.2	Fused silica/Pt
